# Efficacy of Sorafenib Combined with Venetoclax and Azacitidine in Newly Diagnosed FLT3-ITD-Mutant Acute Myeloid Leukemia: Remission Efficacy and Prognostic Influence of Post-Remission Consolidation Therapy

**DOI:** 10.3390/jcm15197576

**Published:** 2026-09-29

**Authors:** Qi Chen, Wenbing Duan, Jinsong Jia, Jing Wang, Xiaosu Zhao, Guorui Ruan, Hongxia Shi, Yingjun Chang, Xiaojun Huang, Hao Jiang

**Affiliations:** 1 Beijing Key Laboratory of Cell and Gene Therapy for Hematologic Malignancies, National Clinical Research Center for Hematologic Disease, Peking University Institute of Hematology, Peking University People’s Hospital, Peking University, Beijing 100044, China; 2 Peking-Tsinghua Center for Life Sciences, Academy for Advanced Interdisciplinary Studies, Peking University, Beijing 100871, China

**Keywords:** acute myeloid leukemia, FLT3-ITD, sorafenib, venetoclax and azacitidine

## Abstract

**Background:** Mutations of the FLT3 gene, particularly internal tandem duplication (FLT3-ITD), define a biologically aggressive AML subtype with historically poor outcomes. Here we retrospectively assessed the clinical performance and tolerability of sorafenib with venetoclax plus azacitidine (VA) as front-line therapy in patients with newly diagnosed FLT3-ITD-mutated AML. **Methods:** Forty-five consecutive patients who received sorafenib plus VA induction were retrospectively analyzed. Post-remission therapy comprised sorafenib-based chemotherapy consolidation, continued sorafenib-VA, or allogeneic hematopoietic stem cell transplantation (allo-HSCT) after consolidation therapy according to clinical indication and guidelines. Composite complete remission (CRc), measurable residual disease (MRD), overall survival (OS), and relapse-free survival (RFS) were assessed. **Results:** The CR/CRi rate was 95.6% (43/45), and the FCM-MRD negativity rate after induction was 90.7%. At a median follow-up of 20.3 months, the 2-year OS and RFS of the entire cohort were 65.3% and 57.3%, respectively. The relapse rate in the patients receiving post-remission allo-HSCT after consolidation therapy was lower. Continued sorafenib-VA achieved a 2-year OS of 57.1% and a 2-year RFS of 64%. Multivariable analysis identified chemotherapy-based post-remission therapy (*p* = 0.016) and detectable MRD after the second consolidation cycle (*p* = 0.004) as independent adverse prognostic factors for RFS. No treatment-related mortality occurred. **Conclusions:** Sorafenib plus VA is a cost-effective, tolerable induction regimen achieving high remission and MRD negativity rates in FLT3-ITD-mutant AML. For transplant-eligible patients, allo-HSCT significantly improves long-term survival; for transplant-ineligible patients, continued sorafenib-VA yields sustained benefit.

## 1. Introduction

Mutations of the FMS-like tyrosine kinase 3 (FLT3) gene are detected in roughly 40% of adults diagnosed with acute myeloid leukemia (AML) and constitute one of the most clinically impactful genetic lesions in this disease [1]. Among FLT3 alterations, internal tandem duplication (FLT3-ITD) accounts for approximately 30% of AML cases overall and is enriched in younger patients (≤60 years), whereas activating mutations in the FLT3 tyrosine kinase domain (FLT3-TKD) are found in roughly 10% [1]. Before the advent of FLT3-directed therapy, individuals harboring these mutations had noticeably lower remission rates and substantially worse long-term outcomes than their FLT3-wild-type counterparts. Population-level analyses from that era showed 3-year overall survival (OS) hovering below 20% irrespective of whether patients were above or below 60 years of age [2].

The combination of small-molecule FLT3 inhibitors with refinements in allogeneic hematopoietic stem cell transplantation (allo-HSCT) has markedly reshaped the outlook of FLT3-mutated AML. However, head-to-head trials showed that adding a FLT3 inhibitor to standard 7 + 3 induction does not by itself translate into a higher complete remission rate when compared with chemotherapy alone [3,4]—the survival benefit comes instead from deeper responses that enable more patients to reach allo-HSCT. Worldwide, four FLT3 inhibitors (sorafenib, midostaurin, gilteritinib, quizartinib) are commercially available, but only sorafenib and gilteritinib are marketed in China. Sorafenib, the first FLT3 inhibitor to gain regulatory approval [5], lacks a formal label for front-line AML; nonetheless, current practice guidelines endorse it for relapsed/refractory FLT3-ITD disease and, more strongly, for post-transplant maintenance [6]. Gilteritinib is licensed for relapsed/refractory FLT3-mutated AML [7], but, in China, its unit price remains a barrier to broad adoption.

Venetoclax, an oral small-molecule BCL-2 inhibitor, paired with the hypomethylating agent azacitidine (AZA), currently defines the standard of care for older or comorbidity-unfit adults with newly diagnosed AML. In the pivotal randomized study that established this regimen, the composite complete response rate (CRc), which was defined as CR plus CRi (complete remission with incomplete blood count recovery), was 66.4%, and the median OS reached 14.7 months, compared with 9.6 months for azacitidine alone [8]. Notably, the CRc in the FLT3-mutated subgroup was 67% (identical to FLT3 wild-type) but had a shorter median OS (12.5 months; only 9.9 months for FLT3-ITD), underscoring that VA alone leaves an unmet need in this biology. Recent early-phase trials and retrospective series combining a second-generation FLT3 inhibitor (gilteritinib or quizartinib) with VA in newly diagnosed FLT3-mutated patients unfit for intensive chemotherapy suggest that this triplet substantially raises remission rates and prolongs survival compared with VA alone [9,10].

However, evidence specifically addressing sorafenib combined with VA in FLT3-ITD-mutant AML is still sparse. Because next-generation FLT3 inhibitors are largely out of reach for many Chinese patients due to cost and limited availability, we retrospectively reviewed our institutional experience with sorafenib + VA as front-line therapy for newly diagnosed FLT3-ITD-mutant AML, aiming to delineate a more accessible therapeutic option for this population.

## 2. Methods

### 2.1. Patients

We retrospectively identified all adults with newly diagnosed FLT3-ITD-mutated AML who received sorafenib plus VA induction at Peking University People’s Hospital between 1 February 2021 and 30 November 2024. The choice of sorafenib plus VA rather than standard intensive induction chemotherapy reflected treating-physician judgment, generally driven by older age or coexisting conditions such as active infection at diagnosis. Diagnosis was confirmed in each case according to the 5th edition of the *WHO Classification of Hematolymphoid Tumours* [11]. Baseline workup uniformly included bone marrow morphology, immunophenotyping by multiparameter flow cytometry, PCR-based fusion-gene screening, G-banded karyotyping, and a next-generation sequencing panel for somatic mutations. The study was performed in accordance with the Declaration of Helsinki and approved by the Ethics Committee of Peking University People’s Hospital.

### 2.2. Detection of FLT3-ITD Mutation

FLT3-ITD was screened by fluorescent PCR with capillary electrophoresis fragment sizing. Genomic DNA was extracted from diagnostic bone marrow samples with a column-based kit and quantified by spectrophotometry. The FLT3 region spanning the juxtamembrane domain (exons 14–15) was amplified using a 5’-FAM-labeled forward primer and an unlabeled reverse primer; thermal-cycling parameters followed the protocol originally described by Meshinchi and colleagues [12]. Amplicons were checked on agarose gels, heat-denatured with formamide and an internal size standard, and resolved on an ABI 3500 genetic analyzer (Thermo Fisher Scientific, Foster City, CA, USA) with POP-7 polymer (Thermo Fisher Scientific, Foster City, CA, USA). GeneMapper software v5.0 (Thermo Fisher Scientific, Foster City, CA, USA) was used to determine fragment sizes and peak areas; any peak longer than the wild-type amplicon was taken as FLT3-ITD-positive. The mutant allelic ratio (AR) was calculated as mutant-peak-area ÷ wild-type-peak-area, with an analytical lower limit of 0.01. Every sample was run in duplicate alongside positive, negative, and no-template controls. Comutation profiling was performed by targeted NGS.

### 2.3. Treatment Regimens

Induction comprised sorafenib 0.4 g orally twice daily on days 1–21 (or 1–28 at the physician’s discretion), venetoclax 100 mg on day 1, 200 mg on day 2, and 400 mg on days 3–21/28, plus subcutaneous or intravenous azacitidine 75 mg/m^2^ on days 1–7. The exact number of sorafenib/venetoclax days was tailored to each patient’s blood counts. When concomitant moderate or strong CYP3A inhibitors (mostly voriconazole in this cohort) were required, venetoclax doses were down-titrated accordingly. After remission, fit individuals were transitioned to sorafenib (0.4 g twice daily, days 1–7) plus high-dose cytarabine (2 g/m^2^ every 12 h, days 1–3) for consolidation; those who were unsuitable for, or declined, intensive chemotherapy continued sorafenib plus VA. For patients harboring concurrent NPM1 mutations, consolidation cycles were delivered sequentially. Allo-HSCT was pursued in any patient with persistently positive MRD, MRD reconversion to positivity after a period of negativity, or frank disease relapse; patients with intermediate- or adverse-risk disease proceeded to allo-HSCT after two consolidation cycles, in accordance with institutional practice.

### 2.4. Definitions

The primary endpoint was the composite complete remission rate (CRc; CR + CRi). Secondary endpoints were MRD negativity rate, OS and RFS. CR required resolution of leukemia-related symptoms, disappearance of circulating blasts, marrow blasts <5%, no extramedullary disease, neutrophils ≥1.0 × 10^9^/L, and platelets ≥100 × 10^9^/L. CRi applied when the hematologic recovery thresholds for CR were not met but the remaining CR criteria were. Patients evaluable for response but who did not meet criteria for complete remission (CR), complete remission with partial hematologic recovery (CRh), complete remission with incomplete hematologic recovery (CRi), morphologic leukemia-free state (MLFS), or partial remission (PR) were classified as having no response (NR). Relapse denoted reappearance of circulating blasts, ≥5% marrow blasts, or any new extramedullary leukemic involvement. MRD negativity after the second consolidation required negative FCM together with a negative FLT3-ITD PCR; in NPM1-positive patients, an additional ≥3-log reduction in NPM1 transcripts was required. OS was measured from diagnosis to death from any cause; RFS was measured from diagnosis to the first of molecular relapse, morphologic relapse, transplantation, or death.

Adverse events (AEs) were graded per the National Cancer Institute Common Terminology Criteria for Adverse Events, version 4.03 (CTCAE v4.03).

### 2.5. Statistics

Statistical analyses and figure preparation were performed in R (R Foundation for Statistical Computing, Vienna, Austria, version 4.6.0), SPSS Statistics version 26.0 (IBM Corp., Armonk, NY, USA), and GraphPad Prism version 9.0 (Graphpad Software, San Diego, CA, USA). Continuous variables were summarized as median (range) and categorical variables as count (percentage); between-group comparisons used Student’s *t*-test, the chi-square, or Fisher’s exact test, as appropriate. Time-to-event endpoints (OS, RFS) were estimated with the Kaplan–Meier method and compared by the log-rank test. A two-sided *p* < 0.05 defined statistical significance.

## 3. Results

### 3.1. Baseline Characteristics

**Figure 1:**
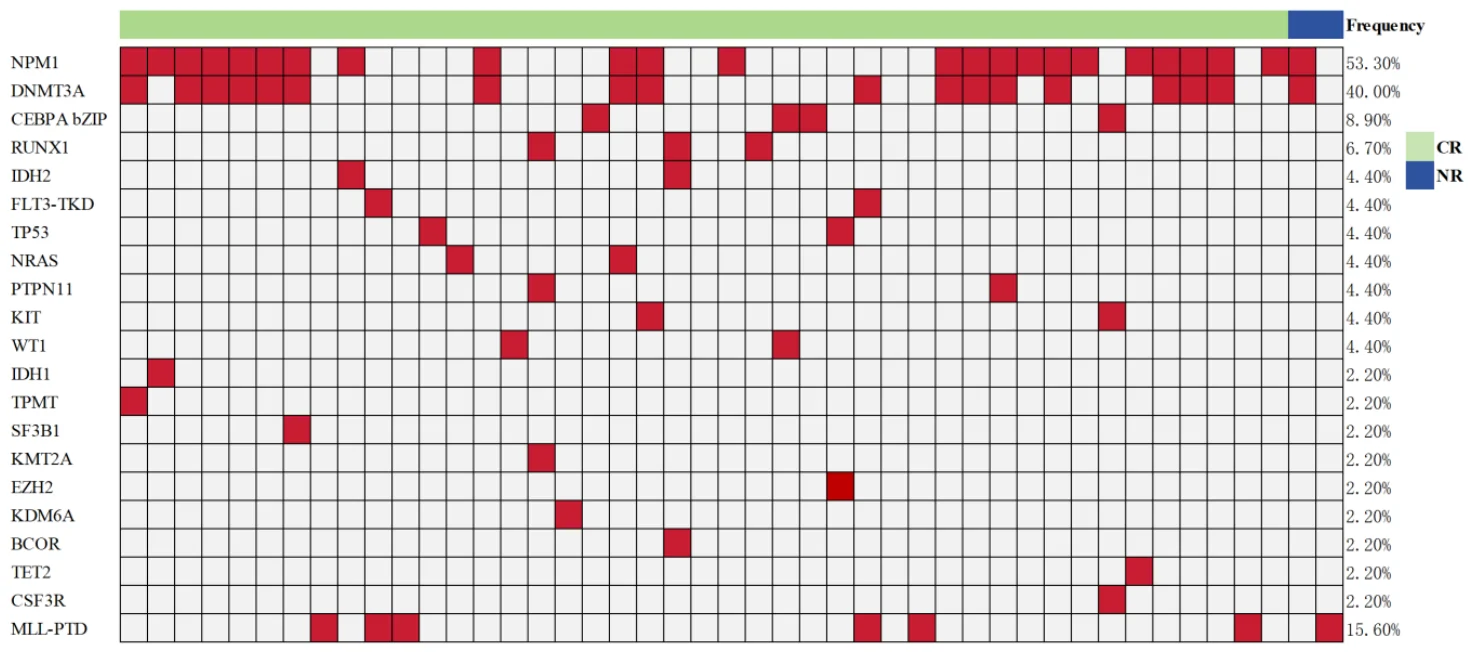
Molecular characteristics of newly diagnosed FLT3-ITD-mutated AML patients treated with sorafenib combined with VA. Each red square indicates an individual patient with a positive gene mutation. CR, complete remission; NR, no response (in accordance with the ELN 2022 criteria, consistent with the definitions in the Methods section).

The baseline characteristics of 45 enrolled patients are summarized in Table 1. The median age of the cohort was 54 years (range, 19–74 years). There were 22 male and 23 female patients. Among all subjects, 20 patients carried FLT3-ITD mutations with a high AR, whereas the remaining 25 had low-AR FLT3-ITD mutations. According to the 2022 ELN risk stratification, 4 patients were classified into the favorable-risk group, 26 into the intermediate-risk group, and 15 into the adverse-risk group. Concurrent gene mutations were identified as follows (Figure 1): NPM1 in 24 patients (53.3%), DNMT3A in 17 (37.8%), IDH1/2 in 4 (8.9%), CEBPA bzip mutation in 4 (8.9%), FLT3-TKD in 2 (4.4%), NRAS in 2 (4.4%), and TP53 in 2 (4.4%). During induction therapy, the median treatment duration of venetoclax combined with sorafenib was 21 days (range, 21–28 days).

### 3.2. Efficacy

All 45 patients received induction treatment with sorafenib plus the VA regimen. A total of 43 patients achieved CR or CRi after a median of 1 cycle (range, 1–2 cycles), translating to a composite complete remission rate of 95.6% (43/45). Forty-two out of forty-three (97.7%) patients achieved CR/CRi after cycle 1, with only one patient achieving CR after cycle 2. The other two patients who failed to achieve CR/CRi had concurrent infections at diagnosis and received induction therapy with VA plus sorafenib. Their infections were controlled after induction, so they switched to sorafenib plus chemotherapy for re-induction treatment. One patient eventually achieved CR, while the other demonstrated no response. Following induction therapy, FCM-MRD turned negative in 39 (90.7%) remission patients, and PCR testing confirmed clearance of an FLT3-ITD mutation in 29 cases (67.4%).

### 3.3. Post-Remission Strategy

**Figure 2:**
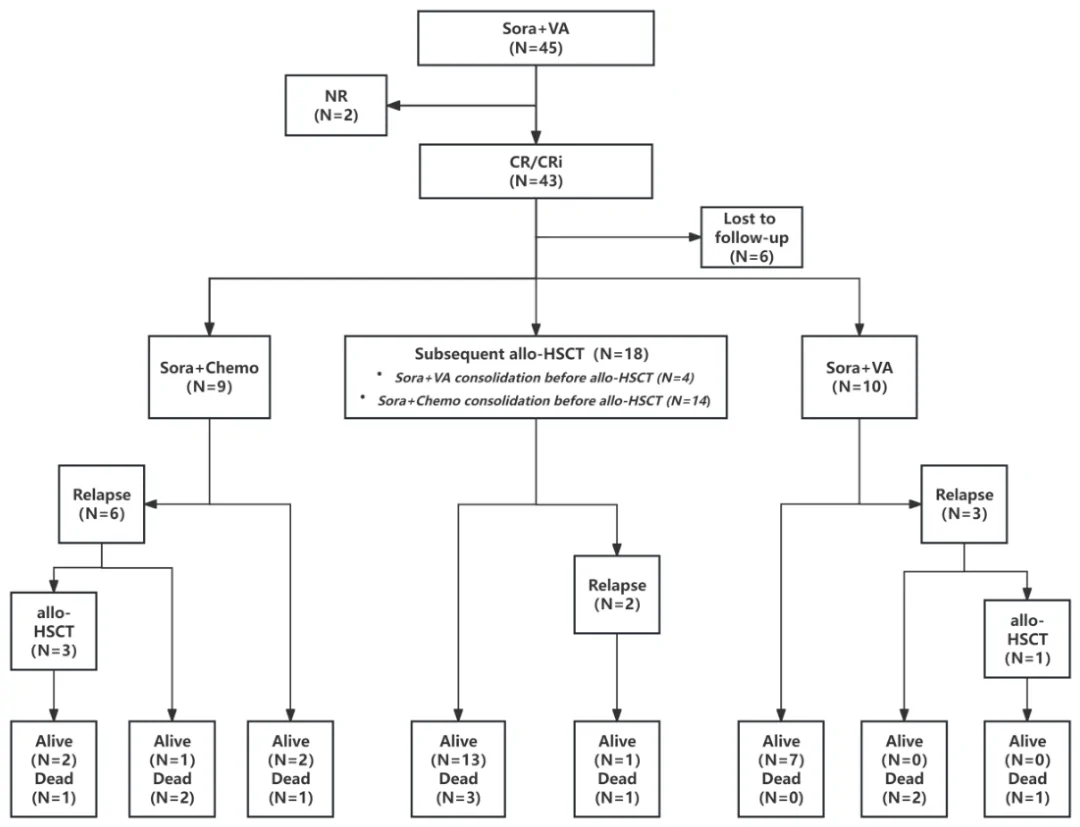
The flowchart of post-remission strategies for newly diagnosed FLT3-ITD-mutated AML patients treated with sorafenib combined with VA.

Among the 43 patients who achieved CR/CRi (Figure 2), 6 were lost to follow-up. Nine (24.3%) patients received sorafenib combined with conventional chemotherapy as post-remission therapy, and 10 (27.0%) maintained treatment with the sorafenib plus VA regimen. The remaining 18 patients (48.6%) underwent subsequent allo-HSCT after consolidation therapy, among whom 4 patients received sorafenib-VA consolidation and 14 received sorafenib-chemotherapy consolidation prior to allo-HSCT. One patient who received VA plus chemotherapy consolidation relapsed after the first cycle of consolidation therapy. Among patients who completed two cycles of consolidation therapy, the MRD negativity rate was 71.4% (10/14) in the sorafenib plus VA consolidation group and 72.7% (16/22) in the sorafenib plus chemotherapy group, with no statistically significant difference between the two cohorts (*p* = 0.932). Among patients with concomitant NPM1 mutations (n = 18), the MRD negativity rate after the second cycle of consolidation therapy was 71.4% (5/7) in those receiving sorafenib combined with VA for consolidation, versus 80% (8/10) in patients treated with sorafenib plus chemotherapy for consolidation, with no significant difference (*p* = 0.682). One patient experienced disease relapse after the first consolidation cycle of sorafenib plus chemotherapy. Baseline clinical characteristics of the three post-remission strategy subgroups are presented in Table S1. Patients in the sorafenib-VA group had a median age of 66 years, which was substantially higher than that of the patients in the sorafenib-chemotherapy group (52 years) and the subsequent allo-HSCT group (39 years) (*p*< 0.010). High-risk cytogenetic and molecular abnormalities accounted for 55.6% of cases in the subsequent allo-HSCT group. By contrast, intermediate-risk stratification was predominant in both the sorafenib-VA group (70.0%) and sorafenib-chemotherapy group (66.7%). The proportion of patients with co-occurring NPM1 mutations was 70.0% (7/10) in the sorafenib-VA cohort, 55.6% (5/9) in the sorafenib-chemotherapy cohort, and 33.3% (6/18) in the subsequent allo-HSCT cohort (*p* = 0.150).

### 3.4. Survival and Relapse

**Figure 3:**
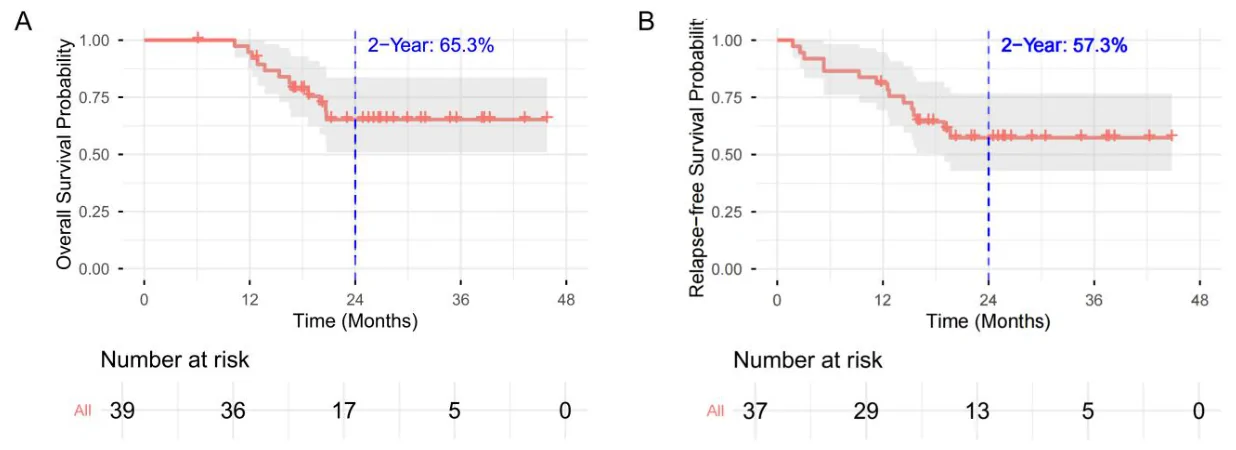
Overall survival (OS) (**A**) and relapse-free survival (RFS) (**B**) in newly diagnosed FLT3-ITD-mutated AML patients treated with sorafenib combined with VA.

**Figure 4:**
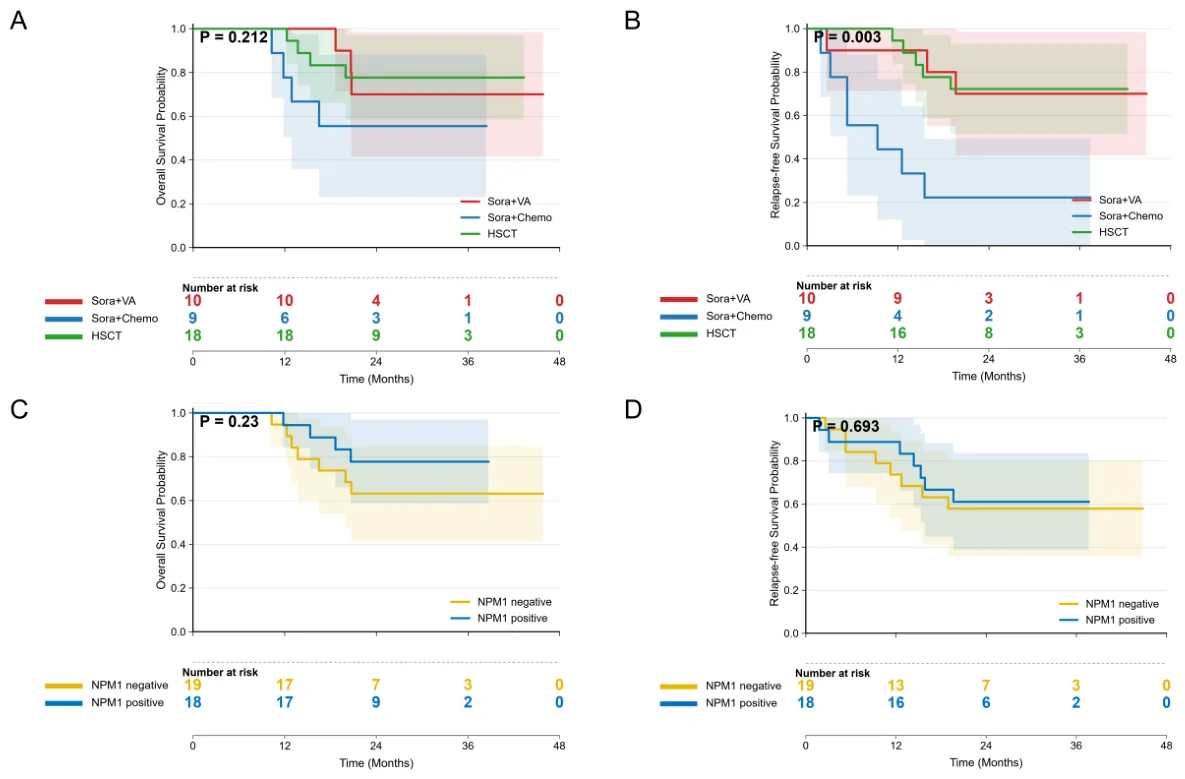
OS (**A**) and RFS (**B**) of FLT3-ITD-mutated AML patients with different post-remission strategies. OS (**C**) and RFS (**D**) of FLT3-ITD-mutated AML patients treated with or without an NPM1 mutation.

Excluding 6 patients lost to follow-up, the median follow-up of the remaining patients was 20.3 (6.1–45.8) months. At a median follow-up of 20.3 months, the median OS for patients who received sorafenib combined with VA for induction was not reached, with the 2-year OS rate being 65.3% (95% CI: 50.9–83.6%, Figure 3A). The median RFS was also not reached, with the 2-year RFS being 57.3% (95% CI: 42.9–76.6%, Figure 3B). For patients receiving different post-remission strategies, the 2-year OS was 57.1% (95% CI: 30.1–100%) for sorafenib combined with VA, 50.0% (95% CI: 25–100%) for sorafenib combined with chemotherapy and 76.3% (95% CI: 58.4–99.7%) for subsequent allo-HSCT (*p* = 0.212, Figure 4A). The 2-year RFS was 22.2% (95% CI: 6.5–75.4%) for sorafenib combined with chemotherapy, 64% (95% CI: 37.4–100%) for sorafenib combined with VA and 70.3% (95% CI: 51.5–96.1%) for subsequent allo-HSCT (*p* = 0.003, Figure 4B). Pairwise comparisons demonstrated a significantly lower relapse rate in the allo-HSCT group. No statistically significant differences were observed between sorafenib-VA and sorafenib-chemotherapy (*p* = 0.093) or between sorafenib-VA versus allo-HSCT (*p* = 0.185, Figure S1). Among the 9 patients in the sorafenib-chemotherapy group, 6 relapsed. Of these, 3 patients harbored NPM1 mutations and all underwent allo-HSCT after achieving remission with re-induction therapy; 1 carried a CEBPA bZIP mutation and received no further treatment after relapse; 1 harbored a TP53 mutation; and 1 had an isolated FLT3-ITD mutation. The latter 2 patients declined transplantation due to financial constraints. Although no statistically significant difference was achieved, patients with an NPM1 mutation exhibited slightly superior OS compared with those without an NPM1 mutation (2-year OS: 75.2% (95% CI: 56.6–99.9%) vs. 56% (95% CI: 35.7–85.8%), *p* = 0.230, Figure 4C). The 2-year RFS rates of patients with an NPM1 mutation and those without an NPM1 mutation were 60.0% (95% CI: 40.8–88.3%) and 55.4% (95% CI: 36.3–86.4%) (*p* = 0.693), respectively. The characteristics of patients with and without an NPM1 mutation are shown in Table S2.

### 3.5. Univariate and Multivariate Analyses

As shown in Table 2, univariate and multivariate prognostic analyses demonstrated that persistent MRD positivity after the second consolidation cycle (HR 0.190, 95% CI 0.060–0.596; *p* = 0.004) and chemotherapy-based post-remission strategy (*p* = 0.016) were independent adverse prognostic factors for RFS in patients who achieved remission following sorafenib combined with VA induction therapy. Compared with sorafenib-VA consolidation, post-remission sorafenib combined with conventional chemotherapy conferred a markedly elevated risk of relapse or death (HR 9.084, 95% CI 1.749–47.178; *p* = 0.009). No significant survival discrepancy was observed between the allo-HSCT group and the sorafenib-VA group (HR 3.266, 95% CI 0.474–22.510; *p* = 0.230).

### 3.6. Safety

Every patient experienced at least one adverse event during induction. Grade ≥3 hematologic toxicities were the most frequent, including thrombocytopenia (84.2%), neutropenia (87.1%), febrile neutropenia (45.7%) and anemia (37.1%). The most common non-hematologic event was infection (28.5%), followed by gastrointestinal symptoms (17.1%). Tumor lysis syndrome was observed in three patients. No deaths were attributable to study treatment.

## 4. Discussion

FLT3 mutations represent one of the most prevalent genetic alterations in AML, and the FLT3-ITD in particular identifies a disease subset with a high rate of relapse and historically dismal survival when treated with conventional chemotherapy alone [1,2,13,14]. The introduction of targeted FLT3 inhibitors has fundamentally changed the front-line management of fit patients: in the phase 3 RATIFY trial, adding midostaurin to standard 7 + 3 induction and consolidation chemotherapy significantly prolonged overall survival (OS) in younger adults with FLT3-mutated AML (median OS 74.7 vs. 25.6 months) [3], and in the QuANTUM-First trial, the addition of quizartinib to intensive chemotherapy improved OS in newly diagnosed FLT3-ITD-mutant AML (median OS 31.9 vs. 15.1 months; HR 0.78, 95% CI 0.62–0.98) [4]. Nevertheless, these regimens improve survival largely by deepening responses, enabling successful consolidation, and reducing relapse, whereas the initial morphologic CR rate itself has not been substantially improved by adding FLT3 inhibitors to intensive chemotherapy [3,4,5]. Randomized trials that added sorafenib to intensive induction similarly failed to demonstrate a statistically significant improvement in CR rate, event-free survival, or OS across the overall cohort or the FLT3-ITD subgroup [5,15]. A proportion of patients therefore still fail to achieve remission with chemotherapy-based FLT3-inhibitor regimens and lose the opportunity to proceed to allo-HSCT. In China, the accessibility of FLT3 inhibitors further shapes clinical practice: second-generation agents have been introduced only recently or are mainly approved/reimbursed for relapsed/refractory disease, and their cost limits broad front-line use, whereas sorafenib, although without a formal AML indication, is widely available and inexpensive and is recommended by both international guidelines and the Chinese AML guidelines for post-transplant maintenance in FLT3-ITD-mutant AML [7,16,17]. There is therefore an unmet need for an induction and post-remission strategy that is both highly effective and accessible to Chinese patients with newly diagnosed FLT3-ITD-mutant AML.

In the present cohort, induction with sorafenib combined with VA achieved a composite CR/CRi rate of 95.6% and an FCM-MRD negativity rate of 90.7% after induction. These figures compare favorably with the benchmark set by VA alone, in which the composite CR rate among FLT3-mutated patients was 67% and the median OS was 12.5 months (9.9 months for the FLT3-ITD subgroup) [18], and are consistent with recently reported FLT3-inhibitor/VA triplets. In a phase I/II study by Short and colleagues, azacitidine, venetoclax, and gilteritinib produced a CR/CRi rate of 96% in 30 treatment-naive patients with FLT3-mutated AML unfit for intensive chemotherapy (median age 71 years; 73% FLT3-ITD), with 18-month relapse-free survival and OS rates of 71% and 72%, respectively [9]. A Chinese retrospective study using the same sorafenib + VA backbone in FLT3-ITD-mutant AML likewise reported encouraging remission [19]. The remission and early MRD clearance rates achieved with sorafenib + VA in our real-world cohort therefore closely mirror those of the gilteritinib-based triplet, suggesting comparable efficacy of sorafenib in this venetoclax-based combination, at a substantially lower financial burden and with better accessibility for Chinese patients.

For FLT3-ITD-mutant AML, allo-HSCT in first complete remission (CR1) remains the most effective post-remission strategy. A meta-analysis of 24 prospective trials confirmed a significant relapse-free survival benefit of allogeneic transplantation for intermediate-risk and adverse-risk AML in CR1 (HR 0.76 and 0.69, respectively) but not for favorable-risk disease [20], and a dedicated analysis of FLT3-ITD-mutant, intermediate-risk patients showed that transplantation in CR1 improved both relapse-free survival and OS irrespective of the FLT3-ITD allelic ratio (3-year OS 46–54% with HSCT vs. 18–24% with chemotherapy) [21]. Consistent with these data, in the present cohort the 18 patients who proceeded to allo-HSCT after consolidation achieved the lowest relapse rate (cumulative incidence of relapse significantly lower than that of the sorafenib-chemotherapy group; *p* = 0.002) and the best 2-year OS and RFS (76.3% and 70.3%, respectively), supporting current guideline recommendations that intermediate- or adverse-risk FLT3-ITD-mutant patients should be considered for transplantation after consolidation [16,17]. Long-term post-transplant sorafenib maintenance further reduces relapse and improves survival in this setting in the randomized phase 3 data of Xuan et al. [22]; this benefit has been corroborated in other real-world cohorts, in which post-transplant sorafenib maintenance significantly lowered the cumulative incidence of relapse (*p* = 0.028) and improved OS (*p* = 0.016), particularly among patients transplanted in CR1 [23]. Of note, the benefit of peri-transplant sorafenib may depend on disease status and on the FLT3-ITD allelic ratio: in a retrospective study of 112 patients with low-allelic-ratio (low-AR) FLT3-ITD-mutant AML who underwent allo-HSCT, sorafenib did not improve OS or RFS in the overall cohort, yet it significantly improved survival in the subgroup transplanted for refractory/relapsed disease [24]. However, not all patients are eligible for transplantation because of age, comorbidity, or the absence of a suitable donor, and for these patients, an effective, well-tolerated, and continuous post-remission strategy is needed. In our cohort, transplant-ineligible patients who continued sorafenib + VA consolidation achieved a 2-year RFS of 64.0% and a 2-year OS of 57.1%, which was markedly better than the 2-year RFS of 22.2% observed with sorafenib plus conventional chemotherapy consolidation. Although no randomized comparison of sorafenib + VA with VA alone is available, the remission and early MRD data of FLT3-inhibitor/VA triplets cited above [9,18,19] support the notion that sustained FLT3 inhibition added to the VA backbone may be particularly beneficial for patients who cannot be consolidated by transplantation. These observations, albeit exploratory because of the small sample size and the non-randomized design, suggest that long-term treatment with sorafenib + VA may serve as a valuable post-remission strategy for transplant-ineligible or transplant-intolerant patients with newly diagnosed FLT3-ITD-mutant AML.

Admittedly, this study is limited by its retrospective design and relatively small sample size from a single institution; prospective multicenter trials are warranted to validate these findings. In addition, the three post-remission strategies were compared in a non-randomized manner, and treatment allocation reflected clinical indication; although age, MRD status, and treatment strategy were entered simultaneously in multivariable Cox models, residual confounding and immortal-time bias cannot be entirely excluded, and these analyses should be regarded as exploratory. The relatively short follow-up and the small subgroup sizes limit the precision of survival estimates, and longer follow-up and larger cohorts are needed. Nevertheless, our real-world data provide high-value localized evidence for optimizing treatment strategies in FLT3-ITD-mutant AML.

In conclusion, this single-center retrospective analysis validates that sorafenib plus VA achieves high remission rates, favorable safety, and superior overall and relapse-free survival in newly diagnosed FLT3-ITD-mutant AML, with efficacy comparable to gilteritinib-based regimens. Furthermore, this combination also achieves a high MRD negativity rate and serves as a well-tolerated bridging strategy to allo-HSCT. Our data further demonstrate that young patients with an FLT3-ITD mutation who undergo allo-HSCT after achieving CR have the best survival outcomes, suggesting that allo-HSCT remains the current optimal curative approach for FLT3-ITD-mutant patients, particularly for those with high-risk features. Moreover, this combination provides sustained survival benefits for transplant-ineligible elderly patients through continuous treatment. Therefore, the combination of VA plus sorafenib represents a treatment regimen that balances efficacy, safety, and cost-effectiveness for FLT3-ITD-mutant AML, particularly in regions where access to FLT3 inhibitors is limited.

## Figures and Tables

**Table 1:** Baseline characteristics of newly diagnosed FLT3-ITD-mutated AML patients treated with sorafenib combined with VA.

	Sora + VA (n = 45)
Median age, years (range)	54 (19–74)
Sex, n (%)	
Male	22 (48.9)
Female	23 (51.1)
FLT3-ITD mutations’ allelic ratio, n (%)	
<0.5	25 (55.6)
≥0.5	20 (44.4)
ELN risk stratification, n (%)	
Favorable risk	4 (8.9)
Intermediate risk	26 (57.8)
Poor risk	15 (33.3)
Co-occurring mutations, n (%)	
NPM1	24 (53.3)
DNMT3A	17 (37.8)
FLT3-TKD	2 (4.4)
IDH1/2	4 (8.9)
KRAS/NRAS	2 (4.4)
CEBPA bZIP	4 (8.9)
TP53	2 (4.4)
MDS-related mutations	5 (11.1)
Cytogenics, n (%)	
Normal karyotype	29 (64.4)
Complex karyotype	4 (8.9)
Additional chromosomal abnormalities	12 (26.7)
Median follow-up, months (range)	20.0 (1.8–45.8)

**Table 2:** Univariate and multivariate analyses of RFS in FLT3-ITD-mutated patients achieving remission with sorafenib combined with VA induction therapy.

Variable	Univariate	Multivariate	
	*p*-Value	*p*-Value	HR, 95% CI
Gender	0.511		
Age	0.864	0.223	1.037 (0.980–1.097)
FLT3-ITD mutation ratio	0.897		
NPM1 mutation status	0.695		
DNMT3A mutation status	0.640		
ELN risk stratification	0.736		
MRD negativity after 2nd consolidation	0.002	0.004	0.190 (0.060–0.596)
Post-remission strategy	0.008	0.016	
Sora + Chemo vs. Sora + VA	0.024	0.009	9.084 (1.749–47.178)
HSCT vs. Sora + VA	0.911	0.230	3.266 (0.474–22.510)

## Data Availability

The data used in this retrospective study were extracted from the electronic medical record system of Peking University People’s Hospital. All patient records were de-identified to remove personally identifiable information. Due to patient privacy protection and institutional ethical restrictions, the raw dataset cannot be publicly shared. The minimal de-identified dataset supporting the findings of this study is available from the corresponding author (email: jiangha0090@sina.com) upon reasonable request and after approval by the institutional ethics committee.

## Supplementary Materials

The following supporting information can be downloaded at: https://www.mdpi.com/article/10.3390/jcm15197576/s1, Figure S1: Cumulative incidence of relapse of different post-remission strategy; Table S1: Baseline characteristics of newly diagnosed FLT3-ITD mutated AML patients with different post-remission strategy; Table S2: Baseline characteristics of newly diagnosed FLT3-ITD mutated AML patients with and without NPM1 mutation.
