# Supplementary material for: Efficacy of Sorafenib Combined with Venetoclax and Azacitidine in Newly Diagnosed FLT3-ITD-Mutant Acute Myeloid Leukemia: Remission Efficacy and Prognostic Influence of Post-Remission Consolidation Therapy
Source: J Clin Med. 2026 Sep 29;15(19):7576. doi: 10.3390/jcm15197576 (PMC13665879; doi:10.3390/jcm15197576)
Supplement: Supplementary file 1 [file jcm-15-07576-s001.zip › jcm-4522155-supplementary.pdf]

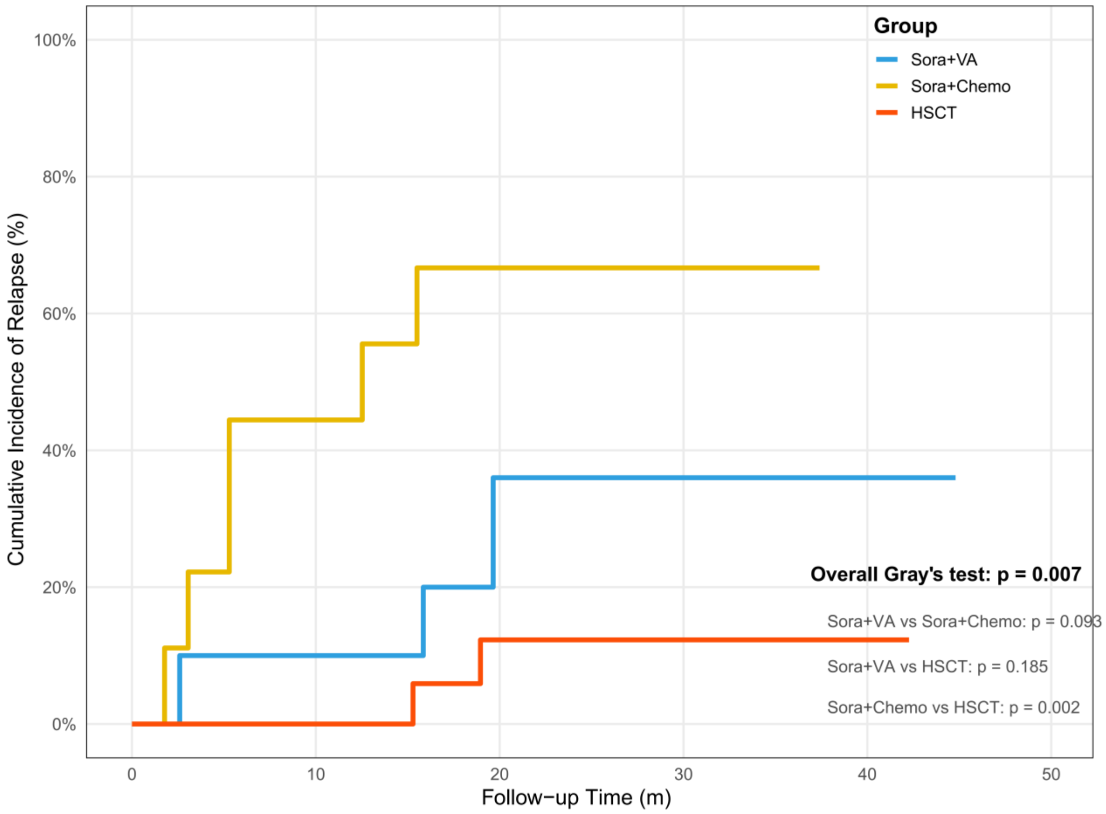


**Supplementary Figure S1** Cumulative incidence of relapse of different post-remission strategy

**Supplementary Table S1** Baseline characteristics of newly diagnosed FLT3-ITD mutated AML patients with different post-remission strategy

| | Sora+VA (N=10) | Sora+Chemotherapy (N=9) | HSCT （N=18） | *p* |
|---|---|---|---|---|
| Median age, years(range) | 66 (54-74) | 52 (39-61) | 39 (19-64) | **<0.01** |
| Sex, n(%) | | | | 0.38 |
| Male | 3 (30) | 5 (55.6) | 10 (55.6) | |
| Female | 7 (70.0) | 4 (44.4) | 8 (55.6) | |
| FLT3-ITD mutations allelic ratio, n(%) | | | | |
| <0.5 | 5 (50.0) | 6 (66.7) | 9 (50.0) | 0.68 |
| ⩾0.5 | 5 (50.0) | 3 (33.3) | 9 (50.0) | |
| ELN risk stratification, n(%) | | | | |
| Favorable risk | 2 (20.0) | 1 (11.1) | 1 (5.6) | 0.13 |
| Intermediate risk | 7 (70.0) | 6 (66.7) | 7 (38.9) | |
| Poor risk | 1 (10.0) | 2 (22.2) | 10 (55.6) | |
| Co-occurring NPM1 mutations, n(%) | 7 (70.0) | 5 (55.6) | 6 (33.3) | 0.15 |
| MRD negativity rate after 2nd consolidation, n(%) | 7(70.0) | 3(33.3) | 16 (88.9) | **0.01** |
| Allo-HSCT, n(%) | 1 (10.0) | 3 (33.3) | 18 (100.0) | **<0.01** |
| Median follow-up, months(range) | 20.6 (17.0-45.8) | 16.5 (6.1-38.4） | 24.2 (12.3-43.3) | 0.33 |

**Supplementary Table S2** Baseline characteristics of newly diagnosed FLT3-ITD mutated AML patients with and without NPM1 mutation

| | NPM1 negative (N=19) | NPM1 positive (N=18) | *p* |
|---|---|---|---|
| Median age, years(range) | 54 (19-68) | 52 (30-74) | |
| Sex, n(%) | | | 0.87 |
| Male | 9 (47.4) | 9 (50.0) | |
| Female | 10 (52.6) | 9 (50.0) | |
| FLT3-ITD mutations allelic ratio, n(%) | | | 0.63 |
| <0.5 | 11 (57.9) | 9 (50.0) | |
| ⩾0.5 | 8 (42.1) | 9 (50.0) | |
| ELN risk stratification, n(%) | | | **<0.01** |
| Favorable risk | 4 (21.1) | 0 (0) | |
| Intermediate risk | 2 (10.5) | 18 (100.0) | |
| Poor risk | 13 (68.4) | 0 (0) | |
| Co-occurring DNMT3A mutation, n(%) | 1 (5.3) | 13 (72.2) | **<0.01** |
| Consolidation therapy | | | 0.16 |
| VA+Sora | 3 (15.8) | 7 (38.9) | |
| Chemotherapy+Sora | 4 21.1) | 5 (27.8) | |
| Allo-HSCT | 12 (63.2) | 6 (33.3) | |
| Relapse, n(%) | 6 (31.6) | 6 (33.3) | 0.64 |
| Median follow-up, months(range) | 21.0 (11.8-38.7) | 20.4 (6.1-45.8) | 0.28 |
